# Effects of polyphenol-rich interventions on the time-course of muscle recovery and athletic performance following exercise-induced muscle damage: a three-level meta-analysis

**DOI:** 10.3389/fnut.2026.1941705

**Published:** 2026-09-04

**Authors:** Xuanming Hu, Haolin Zheng, Jiewen Qiu, Peiyou Chen

**Affiliations:** 1 School of Sports Science and Physical Education, Nanjing Normal University, Nanjing, Jiangsu, China; 2 Business School, Jinling Institute of Technology, Nanjing, Jiangsu, China

**Keywords:** exercise-induced muscle damage, meta-analysis, polyphenols, recovery, supplementation strategy, three-level model

## Abstract

**Background:**

Polyphenol-rich interventions are widely used to support recovery after exercise-induced muscle damage (EIMD), but their effects across outcome domains and recovery periods remain uncertain.

**Methods:**

Seven databases were searched from inception to 14 August 2026 for randomized controlled trials in healthy adults. The primary analysis was restricted to unadjusted post-exercise endpoint values. Three-level meta-analytic models with structured variance-covariance matrices and CR2 cluster-robust inference were used to account for dependent effects. Parallel trials were analyzed using Hedges' g and crossover trials using SMCRP.

**Results:**

Twenty-four randomized controlled trials contributed 195 primary effect sizes. Polyphenol supplementation was associated with a small improvement in pain/soreness (*g* = 0.252, 95% CI 0.049–0.454; *p* = 0.018). Effects on muscle-damage biomarkers (*g* = 0.097, 95% CI −0.249 to 0.442; *p* = 0.555) and performance (*g* = 0.238, 95% CI −0.017 to 0.493; *p* = 0.065) remained uncertain. The perceptual domain was represented by one study and was descriptive only. No statistically detectable differences were identified across prespecified recovery windows (HTZ *p* = 0.480) or supplementation strategies (HTZ *p* = 0.129). Certainty of evidence was low for muscle damage and performance and very low for pain/soreness and perceptual outcomes.

**Conclusion:**

Polyphenol supplementation may modestly reduce post-exercise pain/soreness, but current evidence does not establish consistent improvements in objective recovery, an optimal recovery window, or superiority of any supplementation strategy.

**Systematic review registration:**

https://www.crd.york.ac.uk/PROSPERO/view/CRD420261449278, identifier: CRD420261449278.

## 1 Introduction

Exercise-induced muscle damage (EIMD) is a common physiological response following strenuous or unaccustomed exercise. Its typical manifestations include a temporary decline in muscle strength, delayed onset muscle soreness (DOMS), and elevated levels of indirect markers of muscle damage such as creatine kinase (CK) (1, 2). The short-term decline in muscle strength, soreness, and impaired neuromuscular function caused by EIMD can negatively impact subsequent training quality and athletic performance, thereby increasing the demand for recovery management during consecutive training sessions or competitions. Therefore, accelerating functional recovery following EIMD has consistently been a topic of high interest in the fields of sports nutrition and sports medicine.

In recent years, plant-derived polyphenols have received extensive attention due to their potential redox-modulating and anti-inflammatory properties, as well as biological effects that may influence post-exercise recovery. Polyphenols, or polyphenol-rich foods and extracts—such as tart cherry, curcumin, blueberry, pomegranate, quercetin, and New Zealand blackcurrant—have been widely utilized in sports recovery studies. Their potential roles may involve the regulation of redox homeostasis, Nrf2-related endogenous antioxidant defense, and NF-κB-related inflammatory signaling; however, these mechanisms have yet to be consistently validated in human exercise studies (3, 4). Randomized controlled trials (RCTs) have extensively explored these hypotheses, but results remain inconsistent across different formulations and outcomes. For instance, Hunt et al. (5) found that New Zealand blackcurrant extract alleviated muscle soreness 24–48 h post-exercise, accelerated the recovery of maximal voluntary contraction (MVC), and resulted in lower CK levels at 96 h. Conversely, in a half-marathon study, Costello et al. (6) observed no significant effects of New Zealand blackcurrant extract on the recovery of muscle soreness, fatigue, or jumping performance. Studies on curcumin present similar discrepancies between outcomes and across studies; while Mallard et al. (7) observed reduced pain at 48–72 h post-exercise, recent systematic reviews still conclude that the evidence regarding its effects on muscle damage, inflammation, subjective recovery, and functional performance exhibits marked heterogeneity. Regarding supplementation strategies, there is also no consensus on the comparative efficacy of pre-loading (continuous supplementation for several days prior to exercise) vs. acute supplementation (single or short-term supplementation before or after exercise). Such inconsistencies in the direction and magnitude of effects may stem from variations in intervention timing, polyphenol types, exercise protocols, outcome measures, and—crucially—the selection of evaluation time points (4, 8–11). EIMD-related symptoms, biochemical markers, and functional performances exhibit distinct time dependencies, and the magnitude of change and recovery rates of different indicators are not entirely synchronous. Therefore, relying solely on a single post-exercise time point may fail to adequately capture the effect profile of an intervention across the entire recovery phase (1, 2, 8).

At the level of evidence synthesis, several traditional meta-analyses have attempted to summarize the overall effects of polyphenols on EIMD recovery; however, they present two core methodological limitations (8–10). First, previous reviews have primarily employed conventional fixed-effect or random-effects frameworks, typically pooling data separately by outcome or time point. When a single study contributes multiple correlated effect sizes, failure to explicitly account for the dependency structure among these effect sizes may underestimate the uncertainty of the estimates, artificially narrow confidence intervals, and inflate the risk of Type I errors (8, 12–14). Second, although existing meta-analyses have reported stratified effect estimates across multiple post-exercise time points, these time points are typically examined as separate subgroups or independent meta-analyses. There remains a lack of overall testing for effect differences across various recovery time windows within a unified model that explicitly handles correlated effect sizes from the same study. Thus, temporal information should not be interpreted solely based on whether a specific time point reaches statistical significance; instead, it is necessary to formally test for effect differences between distinct recovery windows while accounting for within-study dependencies.

A three-level meta-analysis allows for the retention of multiple relevant effect sizes meeting primary analysis criteria within the same study, while simultaneously modeling the dependencies among these effect sizes and partitioning the sources of variance into sampling error, within-study variance among effect sizes, and between-study variance (12–14). By further incorporating the recovery time window as a moderator variable, it becomes possible to test whether statistically distinguishable effect differences exist among predefined time windows while accounting for effect size dependency, without the need to treat multiple time points from the same study as independent observations.

Against this background, the present study employs a three-level meta-analytic approach to systematically integrate eligible randomized controlled trials. The primary objective is to evaluate the effects of plant-derived polyphenol interventions on various recovery outcome domains following EIMD, including biochemical markers of muscle damage, pain/soreness, and muscle function and athletic performance; perceptual outcomes supported by only a single study will be reported descriptively. Building upon this, we further explore whether the effects differ statistically according to recovery time windows and supplementation strategies. The overall composite effect across different outcome domains will serve merely as a secondary/exploratory result to provide a general directional reference. We hypothesize that polyphenol interventions may confer beneficial effects on certain EIMD recovery outcomes, though the specific effect patterns across different outcome domains and recovery time windows remain to be empirically tested.

## 2 Methods

### 2.1 Protocol and registration

The protocol for this study has been registered on the PROSPERO International Prospective Register of Systematic Reviews (registration number: CRD420261449278) and was conducted and reported strictly in accordance with the Preferred Reporting Items for Systematic Reviews and Meta-Analyses (PRISMA 2020) guidelines (15, 16).

### 2.2 Search strategy

To ensure the comprehensiveness and systematicity of the literature search, this study systematically searched the following seven electronic databases: PubMed, Embase, Cochrane CENTRAL, Web of Science Core Collection, Scopus, SPORTDiscus (EBSCOhost), and China National Knowledge Infrastructure (CNKI). The final search was executed on August 14, 2026, and the search range for each database covered from its inception to this date, with no language or publication year restrictions applied. In addition, manual backward citation searching was performed on the reference lists of all included studies and relevant systematic reviews and meta-analyses to identify potential eligible studies that might have been missed in the electronic database search.

The electronic search strategy was constructed using Boolean logical operators (17) and revolved around three core concept groups: (1) intervention blocks, including plant-derived polyphenols, polyphenol subclasses, representative polyphenol compounds, and polyphenol-rich natural foods or extracts (e.g., polyphenols, flavonoids, anthocyanins, proanthocyanidins, quercetin, catechins, EGCG, curcumin, resveratrol, tart cherry, pomegranate, blueberry, blackcurrant, propolis, and green tea extract); (2) EIMD/exercise recovery context blocks, including terms such as exercise-induced muscle damage (EIMD), delayed-onset muscle soreness (DOMS), eccentric exercise, muscle damage, muscle soreness, muscle recovery, and muscle fatigue; (3) randomized/controlled trial design blocks, including terms such as randomized/randomized, placebo, controlled trial, RCT, crossover/cross-over, double-blind, and single-blind. The strategy was appropriately adapted for different databases according to their controlled vocabularies, field tags, and search syntax.

To enhance search sensitivity, no mandatory outcome indicator blocks were applied in the final electronic search; that is, specific outcome indicators such as CK, LDH, DOMS, VAS, MVIC, MVC, or CMJ were not required to appear in the title or abstract. Whether specific outcomes met the predefined inclusion criteria was determined during the title/abstract and full-text screening stages, rather than being used as mandatory restrictions in the electronic search. The search platforms, search dates, search limits, number of retrieved records, and full search formulas for each database are detailed in Supplementary File S1. The final search strategy was independently reviewed by a second researcher (Haolin Zheng) prior to execution. The search strategy review records and the completed PRISMA-S checklist are provided in Supplementary File S1.

### 2.3 Eligibility criteria

This study developed inclusion and exclusion criteria based on the PICOS (Population, Intervention, Comparison, Outcomes, Study Design) framework.

#### 2.3.1 Population

Included were healthy adults aged 18–60 years with no current musculoskeletal injuries. No restrictions were placed on sports training backgrounds; participants could include healthy individuals without resistance training experience, university students, recreational athletes, and competitive athletes. Participants were required to complete a specific exercise protocol designed to induce exercise-induced muscle damage (EIMD), such as heavy-load eccentric resistance training, drop jumps, prolonged downhill running, marathon running, or repeated sprint running. Studies involving participants aged < 18 or >60 years, clinical populations with metabolic, cardiovascular, or musculoskeletal diseases, and animal experiments were excluded.

#### 2.3.2 Intervention

Eligible interventions included orally administered plant-derived polyphenols, polyphenolic compounds, or polyphenol-rich natural foods, extracts, and supplements. These included, but were not limited to, tart cherry, pomegranate, curcumin, resveratrol, anthocyanins, quercetin, green tea extract, New Zealand blackcurrant, Brazilian green propolis, and jabuticaba. Permitted supplementation strategies encompassed: (1) pre-loading protocols (continuous supplementation for several days prior to exercise); (2) acute supplementation protocols (a single dose administered before or after exercise); and (3) peri-exercise supplementation protocols (continuous supplementation spanning several days before and after exercise). Acute supplementation was not required to be administered “immediately” before or after exercise.

Multi-component compounded interventions where the independent effects of polyphenols could not be isolated (e.g., polyphenols combined with non-polyphenolic active ingredients possessing independent recovery or anti-inflammatory effects) were excluded. Non-dietary routes of administration were also excluded. For multi-arm trials, only comparisons that allowed for the independent evaluation of polyphenol nutritional interventions meeting the above definitions against an eligible control were extracted; combined intervention arms failing to isolate the independent effects of polyphenols were excluded from the quantitative analysis.

#### 2.3.3 Comparison

Randomized trials employing a matched placebo control were included. The placebo was required to match the intervention in terms of administration form, appearance, and sensory characteristics. For food or beverage interventions, the placebo should also match the energy content as closely as possible while containing no active polyphenolic components. Single-arm studies without a control group and studies using only an active substance with established anti-inflammatory, ergogenic, or recovery-promoting effects as the sole control were excluded.

#### 2.3.4 Outcomes

Original studies were required to report baseline data and data from at least one post-exercise recovery time point, and to report at least one of the following three categories of outcomes: (1) biochemical markers of muscle damage, such as creatine kinase (CK) or lactate dehydrogenase (LDH); (2) subjective recovery indicators, such as delayed-onset muscle soreness (DOMS) or visual analog scale (VAS) scores; (3) indicators of muscle function or athletic performance recovery, such as maximal voluntary isometric contraction (MVIC/MVC), countermovement jump (CMJ), squat jump (SJ), or power output.

Providing data for a single eligible post-exercise recovery time point was sufficient to meet the outcome timing requirement; studies were not strictly required to concurrently report 24 h, 48 h, 72 h, or multiple other recovery time points.

The predefined outcomes were conceptually divided into three main clinical/functional domains: biochemical markers of muscle damage, subjective recovery, and muscle function and athletic performance. To prevent the mixed interpretation of different subjective constructs, the subjective recovery domain was further separated into two categories in the analysis dataset: Pain/Soreness (e.g., VAS, DOMS) and Perceptual (e.g., BORG scale). Consequently, the Outcome_Domain in the statistical analysis comprised four coded categories: Muscle damage, Pain/Soreness, Perceptual, and Performance. Notably, the Perceptual category was contributed by only one independent study; thus, it was reserved for descriptive reporting and was not subjected to confirmatory inference as a primary outcome domain.

#### 2.3.5 Study design

Randomized controlled trials, including both parallel and crossover designs, were included regardless of publication language. Non-randomized trials, cross-sectional studies, observational studies, reviews, or other secondary research were excluded, as were original research reports that failed to provide data suitable for eligibility assessment or quantitative extraction. Duplicate publications or reports with overlapping data were identified at the study level, and only the report most relevant to the predefined analysis and containing the most complete information was retained.

### 2.4 Study selection

Literature screening was conducted in accordance with the PRISMA 2020 guidelines. Initially, all records retrieved from the seven databases were imported into the EndNote reference management software, and duplicates were removed using the software's automated duplicate identification feature. The deduplicated records then proceeded to the title and abstract screening phase.

The screening was conducted independently in two phases. In the first phase, two researchers (Xuanming Hu and Senhui Hou) independently read the titles and abstracts based on the predefined PICOS criteria, excluding records that clearly did not match the research question or inclusion criteria. In the second phase, full texts were sought for reports deemed potentially eligible after the initial screening, and the same two researchers independently performed full-text eligibility reviews. Reports for which full texts could not be obtained were separately recorded as “reports not retrieved” and were not included in the count of exclusions made after full-text retrieval. Reports for which full texts were successfully obtained but did not meet the final PICOS criteria or lacked sufficient data for the predefined quantitative analysis were recorded as full-text exclusions, with a primary reason for exclusion specified for each report.

All full-text eligibility judgments were re-verified against the finalized inclusion and exclusion criteria. Specifically, studies were not excluded merely for reporting only a single post-exercise recovery time point or for lacking a complete continuous follow-up at 24 h, 48 h, and 72 h. Every excluded full-text report and its primary reason for exclusion were documented in the study-level full-text exclusion audit in Supplementary File S2.

**Figure 1:**
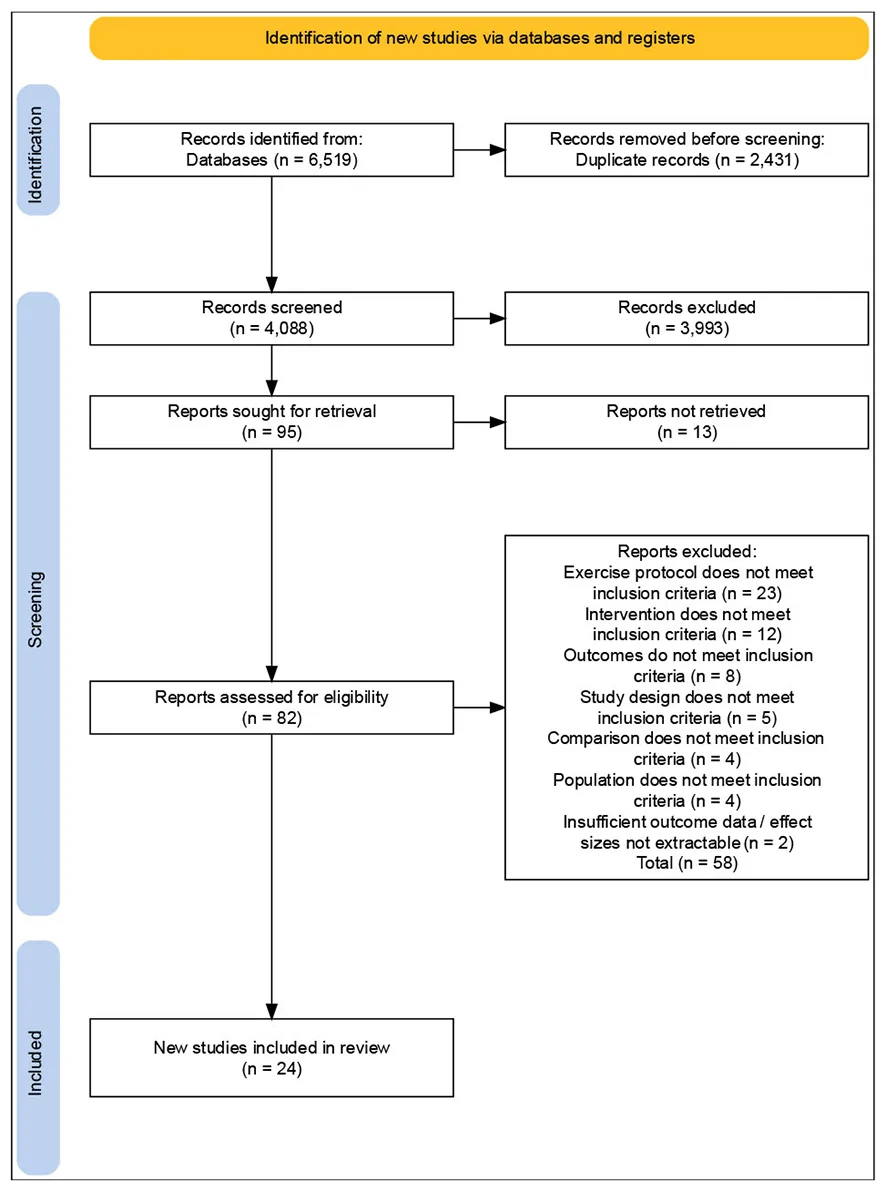
PRISMA flow diagram.

Any disagreements between the two researchers at any screening phase were primarily resolved through discussion. If a consensus still could not be reached, a third researcher (Haolin Zheng) adjudicated and made the final decision. The complete study screening process and the number of records at each stage are presented in a PRISMA 2020 flow diagram in Figure 1.

### 2.5 Data extraction and effect size calculation

Data extraction was performed independently by two researchers using a pre-piloted, standardized data extraction form. Extracted items included basic study information, study design, participant characteristics, sample size, EIMD-inducing protocol, type of polyphenol intervention, dose, supplementation duration, and supplementation strategy. Additionally, group means, standard deviations (SDs), and sample sizes at baseline and each post-exercise measurement time point were extracted for all predefined outcomes. We also recorded the outcome domain, specific outcome indicator, original reporting time, data source (text, table, or graphical extraction via WebPlotDigitizer), and estimand type. For studies reporting values only in graphical format, WebPlotDigitizer was used to extract usable data (18). When necessary information was unavailable from the literature, the original study authors were contacted for retrieval or clarification. If the information required to calculate the effect size and its sampling variance remained unobtainable after author contact and feasible data recovery efforts, the outcome was recorded as unextractable.

Supplementation strategies were independently categorized by two researchers, based on predefined operational definitions, into pre-loading, acute supplementation, and peri-exercise supplementation; any classification disagreements were resolved through discussion. Post-exercise recovery times in the primary analysis were categorized as < 24 h (≥2 h and < 24 h), 24 h, 48 h, 72 h, and 96 h. Observations at 0 h and 1 h post-exercise were not included in the predefined primary analysis time windows or the locked primary analysis dataset; these observations were retained in an independent exploratory dataset and subjected to a *post-hoc* 0–1 h exploratory analysis to evaluate effects during the very early recovery phase. Thus, this exploratory analysis did not alter or enter the primary endpoint-only dataset.

During the final data audit and locking process, each extracted effect estimate was tagged with its estimand type according to its original reporting format. These included: (1) unadjusted endpoint; (2) absolute change from baseline; (3) percentage change from baseline; and (4) percentage of baseline. If the original study provided model-adjusted estimates, they were separately tagged as adjusted estimates. The primary analysis was predefined to exclusively use unadjusted endpoint values, preventing the mixing of endpoint values, absolute change values, percentage change values, or adjusted estimates within the same primary model. Other estimands were kept separate from the primary endpoint dataset and, when data volume permitted, were subjected to independent exploratory or sensitivity analyses; if the number of independent studies was insufficient to support a reliable meta-analysis, they were only reported descriptively and not mixed with the primary endpoint estimates for interpretation.

For parallel-design RCTs, the standardized mean difference for independent groups (Hedges' *g*) was calculated based on the unadjusted endpoint means, SDs, and group sample sizes of the intervention and placebo groups at the same post-exercise time point. Effect sizes and their sampling variances were calculated using metafor::escalc (measure = “SMD”, …) in *R*, with a small-sample bias correction applied (19).

For crossover-design RCTs, since the same participants received both polyphenol and placebo conditions, the standardized mean change using raw-score standardization (SMCRP) was employed. This estimator utilizes the square root of the average of the raw-score variances from the two treatment conditions as the standardizing denominator, rather than using the standard deviation of the change scores (20, 21). Here, M_Exp and M_Pla represent the observed means for the same group of participants under the polyphenol and placebo conditions, respectively, while SD_Exp and SD_Pla are the corresponding raw-score standard deviations. Thus, SMCRP places the treatment condition differences from crossover trials onto a standardized scale based on the dispersion of raw scores.

The small-sample bias correction and the corresponding sampling variance for SMCRP were computed directly by metafor::escalc() (22). The executable call used for the final analysis was:

escalc(

measure = “SMCRP”,

ni = N_Exp,

m1i = Mean_Exp,

sd1i = SD_Exp,

m2i = Mean_Pla,

sd2i = SD_Pla,

ri = Cross_Correlation_Used,

data = data_cross,

append = TRUE

)

Here, ri represents the within-participant correlation between treatment-condition observations (i.e., the correlation between the values observed under the polyphenol and placebo conditions for the same participant). This correlation parameter was used solely for calculating the sampling variance of the SMCRP effect sizes in crossover designs and was not used to construct the V matrix for multiple effect sizes within the same study. When the original study did not report this correlation, the primary analysis assumed an ri of 0.60, and sensitivity analyses were conducted using 0.20, 0.40, and 0.80, respectively.

To unify the direction of effects, the effect sizes for biochemical markers of muscle damage (e.g., CK, LDH, myoglobin) as well as pain/soreness and perceptual indicators (e.g., DOMS, VAS, BORG) were multiplied by −1, so that positive values consistently indicated a favorable recovery effect of the polyphenol intervention. Indicators of muscle function and athletic performance (e.g., MVC, MVIC, CMJ) retained their original direction. Consequently, across all models, a positive effect indicates that the polyphenol intervention has a more favorable recovery direction compared to the placebo.

### 2.6 Risk of bias assessment

The risk of bias of the included studies was assessed using the second version of the Cochrane Risk of Bias tool (RoB 2) (23). The standard RoB 2 tool was applied to parallel-design randomized trials, whereas the RoB 2 variant specific to crossover trials was used for crossover-design randomized trials. Assessments were conducted on a result-specific basis rather than merely at the study level. For each result entering the predefined outcome domain analysis within each study, relevant bias domains were assessed independently. These included the randomization process, deviations from intended interventions, missing outcome data, measurement of the outcome, and selection of the reported result. Based on the RoB 2 algorithm, an overall judgment was generated for that specific result, categorized as “Low risk,” “Some concerns,” or “High risk.”

Two researchers (Xuanming Hu and Senhui Hou) independently completed all RoB 2 assessments. During the assessment process, answers to the signaling questions, the rationale for judgments, and supporting quotes or page numbers from the original literature were recorded. If judgments between the two researchers were inconsistent, a consensus was first sought through discussion; if disagreements persisted, a third researcher (Haolin Zheng) adjudicated. To ensure that the bias judgments corresponded accurately with the specific results in the meta-analysis, each effect size included in the primary analysis was mapped to its outcome-specific RoB 2 judgment, which was then utilized in subsequent risk of bias sensitivity analyses and the GRADE certainty of evidence assessment.

For crossover trials, in addition to formal risk of bias judgments using the crossover-specific RoB 2 tool, potential methodological issues uniquely related to EIMD study designs were explicitly recorded. These included the duration of the washout period, potential carryover effects, period effects, and the potential impact of the repeated bout effect (RBE) (23). These factors were evaluated in conjunction with the specific study design and reported information; a uniform, fixed washout period threshold was not employed as an automatic exclusion criterion, nor were these issues treated as an additional, independent bias domain outside of RoB 2. Instead, their potential impacts were considered as methodological context when interpreting crossover design results.

The complete RoB 2 assessment materials, including answers to signaling questions for both parallel and crossover trials, supporting evidence, domain-level judgments, and outcome-specific overall judgments, are detailed in Supplementary File S2. The risk of bias results were also visually presented using traffic light plots and summary plots.

### 2.7 Statistical analysis

All statistical analyses were conducted in the R 4.3.1 environment, primarily utilizing the metafor package for effect size calculations and multilevel meta-analytic model fitting, alongside the clubSandwich package for cluster-robust inference with CR2 small-sample corrections (22, 24). The methods for effect size calculation, estimand selection, and effect direction unification are detailed in Section 2.5. Primary inferences were based on predefined outcome domain analyses; because the overall composite effect across domains pools biochemical, subjective, and functional outcomes simultaneously, this aggregated result was interpreted merely as a secondary/exploratory analysis to provide a directional reference, rather than as the basis for primary clinical conclusions.

#### 2.7.1 Variance-covariance matrix

To address the dependency among effect sizes arising from multiple outcomes and multiple post-exercise time points within the same study, a study-level block diagonal variance-covariance matrix (V matrix) was constructed using metafor::vcalc() (14, 19). The treatment-condition correlation coefficient (r_cross) used in the calculation of crossover design effect sizes was strictly distinguished from the working correlation parameters in the V matrix: r_cross was exclusively used to calculate the sampling variance of SMCRP, whereas the V matrix utilized independent, structured working correlation parameters to describe the sampling error correlation among multiple effect sizes within the same study.

For the primary analysis, the V matrix was configured with the following working correlation parameters: a within-domain correlation (rho_within) of 0.60, a between-domain correlation (rho_between) of 0.30, and a temporal autocorrelation (phi_time) of 0.60. In vcalc(), Study_ID was specified as the clustering variable, Outcome_Domain was used to distinguish outcome domains, Outcome_Measure was used for specific indicators, and Time_Index represented the temporal structure. The final analysis code explicitly processed r_cross separately from rho_within, rho_between, and phi_time.

For parallel trials containing multiple intervention arms sharing a single placebo group [notably Helder et al. (25) and Lamb et al. (26)], the shared comparison group structure was formally specified using the grp1, grp2, w1, and w2 arguments in vcalc() based on the intervention groups, shared control group, and their respective sample sizes, rather than relying on manual covariance substitution (i.e., 1/N_control). Following construction, the positive definiteness of the V matrix sub-blocks was verified study by study (19). The effect size indices, shared group coding, and corresponding covariance matrix blocks for these two multi-arm studies are detailed in Supplementary File S3.

#### 2.7.2 Three-level random-effects model and heterogeneity partitioning

The predefined outcome domains were analyzed using a three-level random-effects model, with variance components estimated via restricted maximum likelihood (REML). The random-effects structure of the model was specified as ~1 | Study_ID/Effect_Size_ID, thereby partitioning the total variance into: Level 1, the sampling error described by the *V* matrix; Level 2, the residual heterogeneity among different effect sizes within the same study; and Level 3, the between-study heterogeneity (12, 13).

Heterogeneity was described using Cochran's *Q* test, variance components, and the extended *I*^2^ statistic, with the total *I*^2^ further partitioned into within-study heterogeneity (Level 2) and between-study heterogeneity (Level 3). Where estimable, 95% prediction intervals were also reported to reflect the expected range of true effects in real-world study settings (27).

The primary outcome domains included Muscle damage, Pain/Soreness, and Performance. Because the Perceptual category was contributed by only a single independent study, it was reported descriptively, and its individual statistical results were not interpreted as confirmatory meta-analytic evidence.

#### 2.7.3 Cluster-robust inference

All three-level models used Study_ID as the clustering variable and employed cluster-robust variance estimation with CR2 small-sample corrections for inference. Confidence intervals and significance tests for model coefficients utilized small-sample corrected degrees of freedom. The overall differences for moderator variables were evaluated via Wald-type joint tests; under small-sample conditions, the HTZ-corrected results were used as the primary basis for the joint tests, with corresponding Naive-F results retained as a supplement. Predefined between-group comparisons and pairwise comparisons of time windows were executed via linear contrasts of model parameters (28, 29).

#### 2.7.4 Moderator analysis

Predefined moderator variables included: (1) recovery time window: < 24 h, 24 h, 48 h, 72 h, and 96 h; (2) outcome domain: Muscle damage, Pain/Soreness, Perceptual, and Performance; and (3) supplementation strategy: Acute, Peri-exercise, and Pre-loading.

For each moderator variable, a no-intercept model was fitted to obtain independent pooled estimates for each category; whether statistically distinguishable differences existed between categories was evaluated via CR2-corrected joint Wald tests and predefined linear contrasts. For recovery time, in addition to the overall moderator test, all pairwise comparisons among time windows were conducted, with the Holm method applied to adjust for multiple comparisons (30).

Furthermore, to assess the impact of temporal categorization, two alternative time classification analyses were performed: (1) pooling 72 h and 96 h into a “≥72 h” category; and (2) refitting the time model after excluding the 96 h time window entirely.

#### 2.7.5 Small-study effects and publication bias assessment

Given that individual studies contributed multiple correlated effect sizes, traditional Egger's tests based on the assumption of independent observations could be misleading. Therefore, complementary approaches were employed to assess small-study effects (31, 32). First, at the effect-size level, the standard error of the effect size was included as a moderator in the three-level model, applying CR2 robust inference; this analysis served as a reference. Second, correlated effect sizes were aggregated to the study level based on the full V matrix, followed by a study-level Egger regression. The study-level results served as the primary basis for judging overall small-study effects.

Contour-enhanced funnel plots were concurrently used for visual inspection (33). For outcome domain judgments in GRADE, domain-specific small-study effect assessments were further conducted based on the specific set of studies contributing to each domain. Because statistical tests inherently cannot distinguish true publication bias from other small-study effects, the corresponding results were cautiously interpreted as “small-study effects” or “possible publication bias,” rather than as definitive confirmation of publication bias.

#### 2.7.6 Sensitivity and robustness analyses

The following sensitivity and exploratory analyses were conducted to evaluate the dependency of the primary conclusions on analytical specifications; they do not replace the predefined primary outcome domain analyses. Complete numerical results are reported in Supplementary File S4. To test the sensitivity of the results to effect size selection, risk of bias, dependency structures, and model settings, the following predefined and supplementary analyses were implemented:

(1) Leave-one-study-out analysis: sequentially removing each independent study and refitting the model.
(2) Outcome-specific low risk of bias analysis: retaining only those effect sizes whose corresponding specific result was rated as “Low risk” by RoB 2, rather than filtering based solely on an overall study-level RoB rating. The final analysis code was executed conditionally on Outcome_Specific_RoB = = “Low”.
(3) Study design sensitivity analysis: evaluating results when including exclusively parallel RCTs and exclusively crossover RCTs, respectively.
(4) Digital extraction sensitivity analysis: re-analyzing after excluding all observations obtained via WebPlotDigitizer.
(5) V matrix working correlation parameters sensitivity analysis: while maintaining the same structural modeling approach, testing three sets of parameter combinations separately:
  Low: rho_within = 0.20, rho_between = 0.10, phi_time = 0.20;
  Moderate-low: rho_within = 0.40, rho_between = 0.20, phi_time = 0.40;
  High: rho_within = 0.80, rho_between = 0.40, phi_time = 0.80.
(6) Single study-outcome effect size analysis: deterministically retaining one effect size per outcome domain for each study based on predefined temporal and specific-outcome priorities, avoiding the random sampling of time points.
(7) Supplementation strategy classification sensitivity analysis: re-analyzing after excluding Mallard 2020 and Squires 34, which exhibited some ambiguity in their supplementation strategy classifications.
(8) Very early < 24 h data sensitivity analysis: re-analyzing after excluding assessments conducted prior to 4 h post-exercise within the < 24 h window.
(9) Estimand-specific analysis: unadjusted post-exercise endpoints, absolute change from baseline, percentage of baseline, and percentage change from baseline were retained and evaluated separately, and were not mixed with the primary endpoint estimands for interpretation; when an estimand was contributed by only a single study, no independent meta-analytic inference was performed. The primary endpoint dataset contained 195 effect sizes from 24 studies; other estimands were preserved separately.
(10) Crossover design treatment-condition correlation sensitivity analysis: setting the within-participant correlation between treatment-condition observations in the SMCRP calculation to r_cross = 0.20, 0.40, and 0.80, and comparing the results to the primary analysis parameter of 0.60.
(11) Alternative time window classification analysis: re-analyzing after pooling 72 h and 96 h into “≥72 h”, and after excluding the 96 h data.
(12) Shared control covariance sensitivity analysis: comparing the model results obtained by formally specifying the shared control structure via grp1/grp2/w1/w2 against results from a model omitting this specification; in the latter, different intervention arms were treated as independent subgroups, though the outcome and temporal dependency structures within each intervention arm were preserved.
(13) All-pairwise time window comparisons: conducting CR2-corrected pairwise linear contrasts across < 24 h, 24 h, 48 h, 72 h, and 96 h, employing the Holm method to adjust for multiple comparisons.
(14) 0–1 h *post-hoc* exploratory analysis: observations at 0 h and 1 h post-exercise did not enter the locked primary endpoint-only dataset; instead, an independent exploratory dataset was used for 0–1 h pooled and time-specific analyses. This analysis was explicitly marked as a *post-hoc* exploratory analysis and did not enter the locked primary endpoint-only dataset; full results are reported exclusively in Supplementary File S4.
(15) Influence diagnostics: computing Cook's distance and DFBETAS at the study cluster level for the primary models, and integrating these with the leave-one-study-out analysis to identify any individual studies exerting disproportionate influence on the overall results (35).

Except for sensitivity analyses that explicitly altered the *V* matrix structure, all other analyses maintained the three-level random-effects structure and CR2 study-level clustered inference consistent with the primary model. For sensitivity analyses specifically designed to examine the shared control structure or *V* matrix working correlation parameters, only the targeted parameters or structures were modified, while all other model settings remained constant.

#### 2.7.7 Baseline descriptive checks

Pre-exercise baseline data were utilized separately for descriptive checks. A corresponding three-level model was constructed exclusively for baseline effect sizes that yielded calculable, finite, and positive sampling variances; baseline observations lacking valid sampling variances due to zero variance, standardization issues, or other reasons were excluded from this model. Therefore, the baseline analysis does not represent all primary recovery outcomes and serves solely as a descriptive check for post-randomization baseline differences.

Non-significant results in baseline statistical tests were not interpreted as establishing baseline “equivalence” between groups, nor were they used to justify the appropriateness of the unadjusted post-exercise endpoint estimand. The methodological rationale for comparing primary endpoints is fundamentally derived from the random allocation of the original RCTs; the baseline model does not function to attribute causality or test equivalence.

### 2.8 Certainty of evidence assessment

This study adopted the Grading of Recommendations Assessment, Development and Evaluation (GRADE) framework to assess the certainty of evidence for each primary outcome domain (36). Two researchers (Xuanming Hu and Senhui Hou) independently conducted the GRADE evaluations; discrepancies were first resolved through discussion and, when necessary, adjudicated by a third researcher (Haolin Zheng).

GRADE assessments were conducted separately for the four outcome domains: Muscle damage, Pain/Soreness, Perceptual, and Performance. Because the Perceptual domain was contributed by only one independent study, its effect estimates were interpreted primarily descriptively, though its evidence certainty was still independently evaluated according to the GRADE framework. The treatment effect estimates utilized in GRADE were drawn directly from the final primary outcome domain analyses; the overall composite effect across outcomes was not used as a substitute for domain-specific results. Supporting information—including heterogeneity, prediction intervals, risk of bias, participant numbers, and small-study effects—was independently obtained from the specific dataset corresponding to each outcome domain.

The GRADE assessment encompassed five criteria for downgrading evidence certainty:

(1) Risk of bias: evaluated based on the outcome-specific RoB 2 judgments mapped precisely to their corresponding effect sizes, rather than relying on a single overall risk of bias rating for the entire study. The distribution of Low risk, some concerns, and High risk results within the domain and their potential impact on the pooled effect were comprehensively considered.
(2) Inconsistency: judged based on heterogeneity metrics derived from the corresponding outcome domain model, including domain-specific *I*^2^, within- and between-study heterogeneity, 95% prediction intervals, and the consistency of effect directions and magnitudes across studies. Heterogeneity metrics from the cross-outcome overall composite model were not used to substitute judgments for individual outcome domains (37).
(3) Indirectness: assessed by comparing the degree of alignment between the populations, interventions, comparisons, outcomes, and study contexts of the included studies in each domain against the predefined research questions of this systematic review, evaluating the presence of any clinical or methodological differences that might limit the direct applicability of the evidence.
(4) Imprecision: comprehensively judged by considering the 95% confidence intervals of the primary outcome domain effect estimates, whether the confidence intervals crossed the line of no effect, the potential range of important effects, and the optimal information size (OIS) (38, 39). This study pre-specified a standardized mean difference of 0.20 as the target effect size of interest; computing the OIS under conditions of two-sided α = 0.05 and 80% statistical power yielded a required total sample size of 785 participants per outcome domain. The actual total number of participants contributing to each outcome domain was calculated separately and compared against this OIS threshold.
(5) Publication bias: comprehensively judged based on the number of studies in the corresponding outcome domain, domain-specific tests for small-study effects, and visual inspection of funnel plots. Because tests for small-study effects cannot independently prove publication bias, downgrading was only applied when supported by sufficient evidence, deliberately avoiding definitive judgments based on a single statistical test result.

Given that all included studies were randomized controlled trials, the initial certainty of evidence rating for each outcome domain was “High.” Subsequently, based on the five GRADE domains outlined above, judgments were made regarding the presence of “not serious,” “serious,” or “very serious” issues, leading to corresponding downgrades. The final certainty of evidence was ultimately categorized as High, Moderate, Low, or Very low. The GRADE ratings were not automatically generated based on statistical significance; rather, they represented explicit methodological judgments synthesizing the effect estimates, risk of bias, inconsistency, indirectness, imprecision, and publication bias of each outcome domain. The final Summary of Findings and the specific rationale for any downgrades across the GRADE domains are reported in Section 3.8.

## 3 Results

### 3.1 Study selection

The final updated search yielded a total of 6,519 literature records. All retrieved records were imported into the EndNote reference management software for deduplication. A total of 2,431 duplicate records were removed, leaving 4,088 unique records to advance to the title and abstract screening phase. After completing the initial screening based on the predefined PICOS inclusion and exclusion criteria, 3,993 records were excluded, and 95 reports proceeded to the full-text retrieval phase.

Of the 95 reports sought for full-text retrieval, full texts for 13 could not be obtained; these were documented separately as “reports not retrieved” and were not included in the subsequent full-text exclusion tally. Ultimately, 82 reports were successfully retrieved in full text and underwent eligibility assessment.

Following a comprehensive review of all obtainable full texts against the finalized inclusion and exclusion criteria, 58 reports were excluded. To avoid double counting, each excluded report was assigned only one primary reason for exclusion. The specific reasons for exclusion were as follows: exercise protocols not meeting inclusion criteria (*n* = 23); interventions not meeting inclusion criteria (*n* = 12); outcome measures not meeting inclusion criteria (*n* = 8); study design or publication type not meeting inclusion criteria (*n* = 5); comparators not meeting inclusion criteria (*n* = 4); populations not meeting inclusion criteria (*n* = 4); and insufficient outcome data precluding the calculation of effect sizes and their sampling variances under the predefined effect size framework (*n* = 2).

During the re-evaluation process, studies were not excluded merely for reporting a single eligible post-exercise recovery time point or for lacking consecutive 24 h, 48 h, and 72 h follow-ups. Reports that could not be retrieved were recorded separately from those excluded after full-text assessment. An itemized list of the excluded full-text reports, along with their primary reasons for exclusion, is provided in the study-level full-text exclusion list in Supplementary Materials.

Ultimately, 24 randomized controlled trials met all inclusion criteria and proceeded to the quantitative synthesis. The complete study selection process and the number of records at each stage are illustrated in the PRISMA 2020 flow diagram (Figure 1).

### 3.2 Study characteristics

The 24 included randomized controlled trials comprised 17 parallel-design trials and 7 crossover-design trials (5, 7, 25, 26, 34, 40–56, 62, 63). The sources of polyphenol interventions varied widely, with tart cherry (9 studies) and curcumin (5 studies) being the most prevalent. The remaining interventions involved other polyphenol-rich natural foods, extracts, or supplements, including jabuticaba pulp, Brazilian green propolis, quercetin, New Zealand blackcurrant, blueberry, pomegranate, blackcurrant, and bilberry.

Based on the predefined classification of supplementation timing, 11 studies were categorized as utilizing pre-loading, 9 as peri-exercise supplementation, and 4 as acute supplementation. Pre-loading primarily refers to protocols involving continuous supplementation for several days prior to exercise; peri-exercise supplementation indicates continuous supplementation spanning multiple periods both before and after exercise; and acute supplementation denotes a single or short-term dose administered primarily immediately before or after exercise. To avoid classifying supplementation strategies based solely on the total number of supplementation days, the actual durations of supplementation prior to, on the day of, and post-exercise for each study are explicitly detailed in Table 1.

**Table 1:** Characteristics of included studies.

Study	Design	Sample^*^	Polyphenol intervention	EIMD protocol	Main outcomes	Time points	Pre-load (d)	Ex-day	Post-load (d)	Total (d)
Squires et al. (34)	Parallel	35 recreationally trained M/F marathon runners	Tart cherry (pre-load 7 d)	Marathon run	MVC, CMJ, DOMS, CK	Pre, 24 h, 48 h	4	1	2	7
Ramos Junior et al. (54)	Parallel	22 resistance-trained F	Propolis (Artepillin C, pre-load 7 d)	Eccentric knee extensions	MVIT, DOMS	Pre, 2 h, 24 h, 48 h, 72 h	3	1	3	7
Junior et al. (62)	Parallel	24 resistance-trained M/F	Jaboticaba juice (~1,300 mg polyphenols, pre-load 7 d)	Eccentric elbow flexion	MVCisok, DOMS	Pre, 2 h, 24 h, 48 h, 72 h	3	1	3	7
Mallard et al. (21)	Parallel	27 recreationally trained M	Curcumin (427 mg curcuminoids, acute multiple, 4 d)	Leg press to exhaustion	CK, LDH, Myoglobin	Pre, 3 h, 24 h, 48 h, 72 h	0	1	3	4
Squires et al. (34)	Parallel	22 recreationally active M/F	Tart cherry (acute post-exercise, 4 d)	Eccentric elbow flexors	MVC, DOMS	Pre, 24 h, 48 h, 72 h	0	1	3	4
Hunt et al. (5)	Parallel	27 non-resistance trained M/F	NZ blackcurrant (105 mg anthocyanins, pre-load 12 d)	Eccentric elbow flexors	MVC, CK, DOMS	Pre, 24 h, 48 h, 72 h, 96 h	7	1	4	12
Bazzucchi et al. (49)	Crossover	16 active M	Quercetin (1,000 mg/d, pre-load 14 d)	Eccentric elbow flexion	DOMS	Pre, 24 h, 48 h, 72 h, 96 h	14	1	0	15
Helder et al. (25)	Parallel	33 recreationally active M (LOW 11, HIGH 12, PLA 10)	Curcumin 750/1,500 mg/d (pre-load 7 d)	Leg press eccentric	CK, VAS, BORG, Peak Torque	Pre, 24 h, 48 h, 72 h	2	1	4	7
Junior et al. (63)	Parallel	24 resistance-trained M/F	Jaboticaba juice (~1,060 mg polyphenols, pre-load 7 d)	Eccentric knee extensions	MVIC, DOMS	Pre, 2 h, 24 h, 48 h, 72 h	3	1	3	7
Ghojazadeh et al. (52)	Parallel	18 trained M taekwondo athletes	Curcumin (turmeric extract, pre-load 6 d)	Simulated taekwondo competitions	CK, LDH	Pre, 24 h, 48 h	3	1	2	6
Nieman et al. (53)	Parallel	49 healthy M/F	Blueberry powder (805 mg phenolics, pre-load 18 d)	90-min eccentric exercise bout	DOMS, CK	Pre, 24 h, 48 h, 72 h, 96 h	14	1	4	19
Morehen et al. (50)	Crossover	11 professional M rugby players	Tart cherry (Montmorency, pre-load 8 d)	Rugby match	DOMS, CMJ, DJ (RSI)	Pre, 24 h, 48 h	5	1	2	8
Difranco et al. (51)	Parallel	39 endurance-trained M marathoners	Tart cherry (Montmorency, pre-load 8 d)	Marathon run	CK, MVIC	Pre, 24 h, 48 h	5	1	2	8
Nicol et al. (44)	Crossover	17 healthy active M	Curcumin (5 g/d, pre-load 6 d)	Eccentric leg press	DOMS, CK	Pre, 24 h, 48 h	2.5	1	2.5	6
Tanabe et al. (45)	Crossover	14 healthy untrained M	Curcumin (Theracurmin, acute, 1 d)	Eccentric elbow flexors	MVC, ROM, DOMS, CK	Pre, 24 h, 48 h, 72 h, 96 h	0	1	0	1
Trombold et al. (41)	Crossover	16 recreationally active M	Pomegranate extract (650 mg polyphenols, pre-load 9 d)	Eccentric elbow flexion	Isometric strength, Soreness, CK, Myoglobin	Pre, 2 h, 24 h, 48 h, 72 h, 96 h	4	1	4	9
Lamb et al. (26)	Parallel	36 non-resistance trained M	Tart cherry or Pomegranate (pre-load 9 d)	Eccentric elbow flexors	MIVC, DOMS, ROM, CK	Pre, 24 h, 48 h, 72 h, 96 h	4	1	4	9
Hutchison et al. (43)	Parallel	16 untrained M/F college students	Black currant nectar (anthocyanins, pre-load 8 d)	Eccentric squats	DOMS, CK	Pre, 24 h, 48 h, 96 h	4	1	3	8
Bell et al. (46)	Parallel	16 semi-professional M soccer players	Tart cherry (Montmorency, pre-load 8 d)	Soccer match simulation	Agility, Sprint, DOMS, CK	Pre, 24 h, 48 h, 72 h	4	1	3	8
Howatson et al. (40)	Parallel	20 recreational M/F marathon runners	Tart cherry (Montmorency, pre-load 8 d)	Marathon	CK, LDH, DOMS, MVIC	Pre, 24 h, 48 h	5	1	2	8
Lynn et al. (48)	Parallel	19 recreationally trained M/F runners	Bilberry juice (pre-load 8 d)	Half-marathon	DOMS	Pre, 24 h, 48 h	5	1	2	8
Rezaei et al. (55)	Crossover	14 collegiate M volleyball players	Pomegranate juice (acute, 1 d)	200 maximal vertical jumps	DOMS, VJH, MVIC	Pre, 12 h, 24 h, 48 h	0	1	0	1
Bowtell et al. (42)	Crossover	10 well-trained M athletes	Tart cherry (Montmorency, pre-load 10 d)	Eccentric single-leg knee extensions	MVC, CK	Pre, 24 h, 48 h	7	1	2	10
Beals et al. (47)	Parallel	29 recreationally active M/F	Tart cherry beverage (freeze-dried, pre-load 12 d)	Repetitive eccentric quadriceps	VAS, AvgPKBW	Pre, 24 h, 48 h, 96 h	4	1	7	12

^*^Sample size is reported as the number of participants analyzed; randomized totals are available in the original reports. CK, creatine kinase; CMJ, countermovement jump; DJ (RSI), drop jump reactive strength index; DOMS, delayed-onset muscle soreness; LDH, lactate dehydrogenase; M/F, males/females; MVC, maximal voluntary contraction; MVCisok, isokinetic maximal voluntary contraction; MVIC, maximal voluntary isometric contraction; MVIT, maximal voluntary isometric torque; PLA, placebo; VAS, visual analog scale; VJH, vertical jump height; BORG, rating of perceived exertion; ROM, range of motion. For Lamb et al. (26), the tart cherry and pomegranate intervention arms were each compared with a shared placebo group. For Helder et al. (25), the low-dose and high-dose curcumin intervention arms were each compared with a shared placebo group. For Difranco et al. (51), only the cherry juice vs. placebo comparison was included in the quantitative synthesis. “Pre-load (d)” indicates the number of supplementation days before the EIMD-inducing exercise; “Ex-day” indicates whether supplementation was administered on the exercise day; “Post-load (d)” indicates the number of post-exercise supplementation days; and “Total (d)” indicates the reported total supplementation duration. Supplementation-strategy classification (Pre-loading, Acute, or Peri-exercise) followed the pre-specified operational definitions described in the Methods and was based on the complete supplementation-timing pattern rather than on any single timing column.

The exercise-induced muscle damage (EIMD) protocols encompassed various exercise modalities, including laboratory-based eccentric resistance training, endurance running (e.g., half or full marathons), and team sports or simulated matches. All included studies reported at least one of the three predefined outcome categories (pain/soreness, biochemical markers of muscle damage, or muscle function and athletic performance) and provided data for baseline and at least one post-exercise recovery time point. The post-exercise assessment time points utilized for the primary endpoint-only analysis ranged from approximately 2 h to 96 h.

For multi-arm trials, only comparisons allowing for the independent evaluation of eligible polyphenol interventions against a matched placebo were included. For instance, in the study by Difranco et al. (51), only the comparison between the cherry juice (CJ) group and the placebo (PL) group was included; the cold-water immersion arm and the combined intervention arm, which precluded the isolation of the independent effects of cherry juice, were excluded from the quantitative analysis. Detailed characteristics of all included studies are summarized in Table 1.

### 3.3 Outcome-specific risk of bias

The 24 included randomized controlled trials were assessed for risk of bias using the Cochrane RoB 2 tool. The standard RoB 2 tool was used for parallel-design trials, while the RoB 2 for crossover trials was used for crossover-design trials. Formal risk of bias judgments were made for specific results and were mapped one-to-one with the Effect_Size_ID in the primary analysis, rather than merely assigning a single overall risk rating to each study. The complete answers to signaling questions, supporting quotes or page numbers, domain-level judgments, and outcome-specific overall judgments are detailed in Supplementary File S2. Among the 195 locked primary endpoint effect sizes, the Muscle damage domain included 65 effect sizes, of which 40 were rated as Low risk and 25 as Some concerns; the Pain/Soreness domain included 83 effect sizes, of which 31 were Low risk and 52 were Some concerns; the Perceptual domain included 6 effect sizes, all derived from the same study and all rated as Some concerns; the Performance domain included 41 effect sizes, of which 6 were Low risk and 35 were Some concerns. No effect sizes in the primary analysis dataset were rated as High risk. The distribution of RoB 2 judgments across primary outcome domains is presented in Table 2. Given the differences in risk of bias distribution across outcome domains, subsequent evidence interpretation and GRADE assessments were all based on the outcome-specific RoB 2 judgments corresponding one-to-one with the primary endpoint effect sizes, avoiding the use of a single study-level overall rating as a substitute for result-specific risk judgments. For crossover trials, design-related issues such as washout periods, potential carryover effects, period effects, and the repeated bout effect were also considered in accordance with the requirements of the RoB 2 for crossover trials; the relevant signaling questions, supporting evidence, and specific judgments are detailed in Supplementary File S2. The outcome-specific RoB 2 judgments were concurrently utilized for the low risk of bias robustness analysis. However, this analysis was strictly used to test the sensitivity of the main results to risk of bias specifications and did not replace the locked primary endpoint analysis; its complete numerical results are reported in Supplementary Materials.

**Table 2:** Outcome-specific risk of bias summary for the primary endpoint analysis.

Outcome domain	Studies	Primary effect sizes	Low risk	Some concerns	High risk
Muscle damage	13	65	40	25	0
Pain/Soreness	20	83	31	52	0
Perceptual	1	6	0	6	0
Performance	12	41	6	35	0
Overall primary dataset	24^*^	195	77	118	0

**Figure 2:**
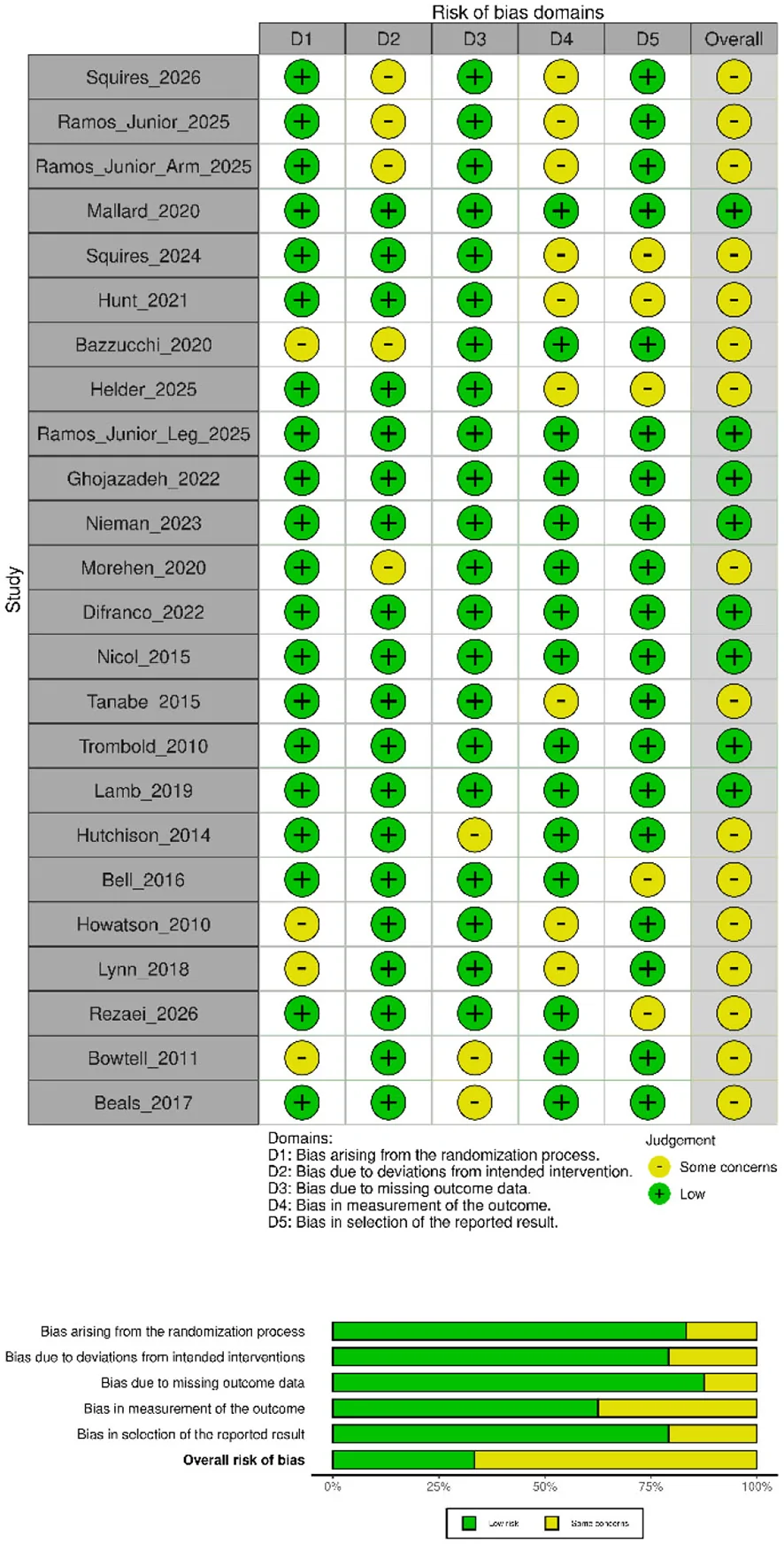
Risk of bias assessment for all included studies using the RoB 2 tool, including a traffic light plot and a summary bar plot.

Risk-of-bias judgments were result-specific and were linked one-to-one to the corresponding primary effect sizes using Effect_Size_ID. Parallel-group trials were assessed using the standard Cochrane RoB 2 tool, whereas crossover trials were assessed using the dedicated RoB 2 for crossover trials. Low, low risk of bias; Some concerns, some concerns regarding risk of bias; High, high risk of bias. Studies may contribute effect sizes to more than one outcome domain; therefore, study counts across outcome domains are not additive. Complete signaling-question responses, supporting quotations/page references, domain-level judgments (D1–D5), and overall result-specific judgments are provided in Supplementary File S2. Figure 2 provides a graphical summary of the outcome-specific RoB 2 assessments. ^*^The overall primary dataset included 24 unique studies. Individual studies may contribute effect sizes to more than one outcome domain; therefore, study counts across outcome domains are not additive.

### 3.4 Baseline descriptive check

Pre-exercise baseline data were utilized solely for descriptive checks and did not constitute part of the primary treatment effect analysis. Because some standardized or normalized baseline records exhibited zero variance or lacked calculable valid sampling variances, not all outcomes entering the post-exercise recovery analysis could be included in the baseline model; thus, this analysis does not represent all primary outcomes. The results of the baseline checks were neither interpreted as establishing baseline “equivalence” between the intervention and placebo groups, nor used to justify the appropriateness of adopting the unadjusted post-exercise endpoint as the primary estimand. The validity of the primary between-group comparisons was fundamentally established on the random allocation of the original randomized controlled trials. Complete results of the baseline descriptive analysis are provided in Supplementary File S4.

### 3.5 Principal outcome-domain analyses

**Figure 3:**
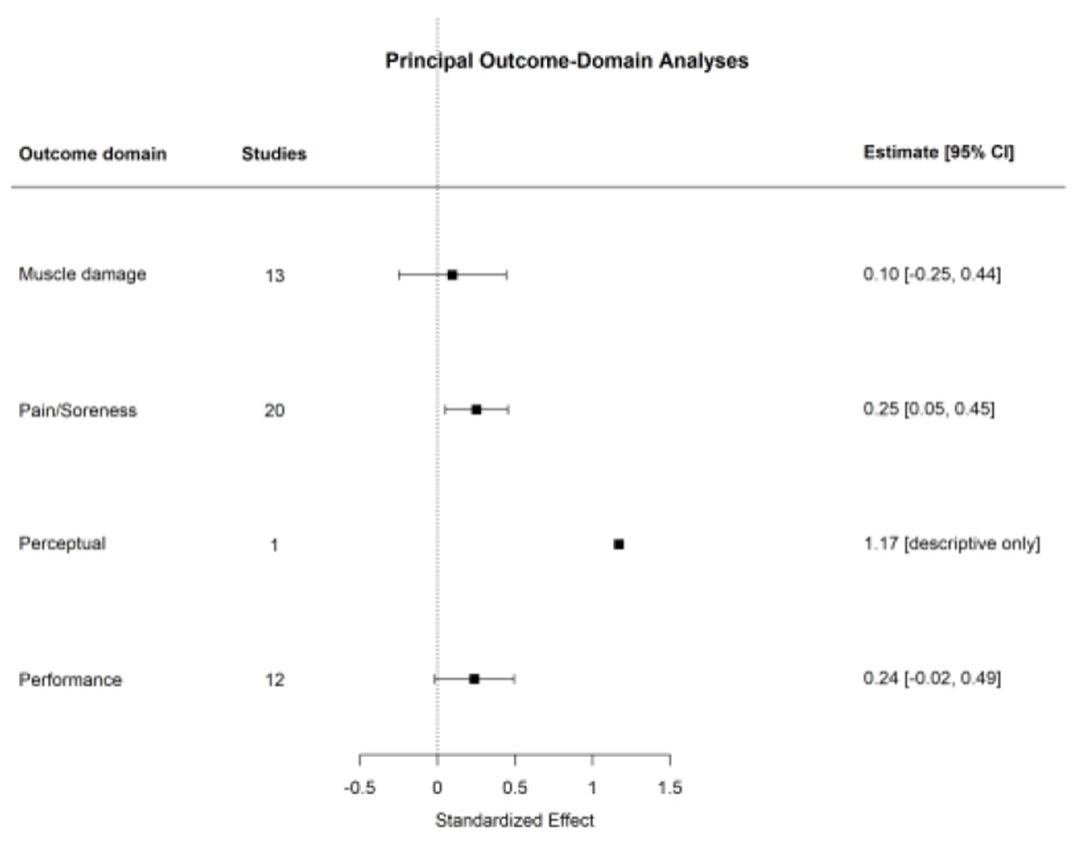
Forest plot of the principal outcome-domain analyses.

The primary analysis was restricted to unadjusted post-exercise endpoints, encompassing a total of 195 effect sizes from 24 independent randomized controlled trials. Primary inferences were conducted separately according to predefined outcome domains, which included Muscle damage, Pain/Soreness, and Performance; the Perceptual domain, being contributed by only one independent study, was reported merely descriptively. The effect estimates and their 95% confidence intervals for each primary outcome domain are presented in Table 3 and Figure 3. For Pain/Soreness, 20 studies contributed a total of 83 effect sizes. Polyphenol interventions were associated with lower post-exercise pain/soreness, with a pooled effect size of *g* = 0.252 (95% CI: 0.049–0.454, CR2 *p* = 0.018), indicating a favorable effect of small magnitude. For Muscle damage, 13 studies contributed 65 effect sizes, yielding a pooled effect size of *g* = 0.097 (95% CI: −0.249 to 0.442, CR2 *p* = 0.555). The confidence interval crossed the line of no effect; thus, current evidence cannot determine whether polyphenol interventions yield meaningful improvements in biochemical markers of muscle damage. For Performance, 12 studies contributed 41 effect sizes, with a pooled effect size of *g* = 0.238 (95% CI: −0.017 to 0.493, CR2 *p* = 0.065). Although the direction of this estimate favored polyphenol interventions, the confidence interval crossed the line of no effect; therefore, uncertainty remains regarding the impact on the recovery of muscle function and athletic performance. The Perceptual domain was contributed by only one independent study (25), which provided 6 effect sizes with a descriptive point estimate of *g* = 1.171. Due to the lack of multiple independent studies, robust CR2 meta-analytic inference could not be performed; hence, this result is reported purely descriptively and does not constitute a confirmatory conclusion.

**Table 3:** Principal outcome-domain meta-analysis results.

Outcome domain	Studies	Effect sizes (*k*)	Standardized effect (95% CI)	CR2 *p*	*I*^2^ total	95% prediction interval
Muscle damage	13	65	0.097 (−0.249, 0.442)	0.555	73.30%	−1.236 to 1.641
Pain/Soreness	20	83	0.252 (0.049, 0.454)	0.018	48.40%	−0.516 to 0.962
Perceptual	1	6	1.171^†^	—	—	—
Performance	12	41	0.238 (−0.017, 0.493)	0.065	15.00%	−0.112 to 0.685

Estimates are based on the locked primary dataset of unadjusted post-exercise endpoint values. All inferential estimates were obtained using three-level meta-analytic models with CR2 cluster-robust inference. Positive values indicate effects favoring polyphenol supplementation. *I*^2^ and prediction intervals were estimated separately within each outcome domain as supporting heterogeneity diagnostics.^†^The Perceptual domain was represented by one independent study; therefore, the point estimate is descriptive only and CR2 inferential statistics, heterogeneity, and prediction intervals were not estimable. Parallel-group effects were calculated using Hedges' g, whereas crossover effects were calculated using SMCRP.

### 3.6 Heterogeneity and prediction intervals of principal outcomes

The degree of heterogeneity varied across primary outcome domains. For the Muscle damage domain, the total I^2^ was 73.3%, with a within-study heterogeneity *I*^2^ of 52.9% and a between-study heterogeneity *I*^2^ of 20.5%; the 95% prediction interval ranged from −1.236 to 1.641. For the Pain/Soreness domain, the total *I*^2^ was 48.4%, with a within-study heterogeneity *I*^2^ of 35.5% and a between-study heterogeneity *I*^2^ of 12.9%; the 95% prediction interval ranged from −0.516 to 0.962. For the Performance domain, the total *I*^2^ was 15.0%, with a within-study heterogeneity *I*^2^ of 15.0% and between-study heterogeneity approaching zero; the 95% prediction interval ranged from −0.112 to 0.685. Because the Perceptual domain was contributed by only a single independent study, between-study heterogeneity and 95% prediction intervals could not be estimated.

### 3.7 Secondary and exploratory analyses

As a secondary analysis, the 195 primary endpoint effect sizes were pooled across all outcome domains into an overall composite effect, yielding a pooled effect size of *g* = 0.215 (95% CI: 0.045–0.385, CR2 *p* = 0.016) with a 95% prediction interval of −0.545 to 0.974. Because this analysis simultaneously integrated biochemical, subjective, and functional outcomes, it is reported solely as a secondary/exploratory result. The overall moderating effect of recovery time was not statistically significant (HTZ *p* = 0.480), indicating no statistically significant differences in effects across the five predefined time windows (< 24 h, 24 h, 48 h, 72 h, and 96 h). Specific effect estimates for each time window, pairwise comparisons, and alternative time classification analyses are detailed in Supplementary File S4. Similarly, the overall moderating effect among different supplementation strategies did not reach statistical significance (HTZ *p* = 0.129). Specific effect estimates and direct between-group comparisons for the pre-loading, acute, and peri-exercise subgroups are presented in Supplementary File S4.

### 3.8 Certainty of evidence

**Figure 4:**
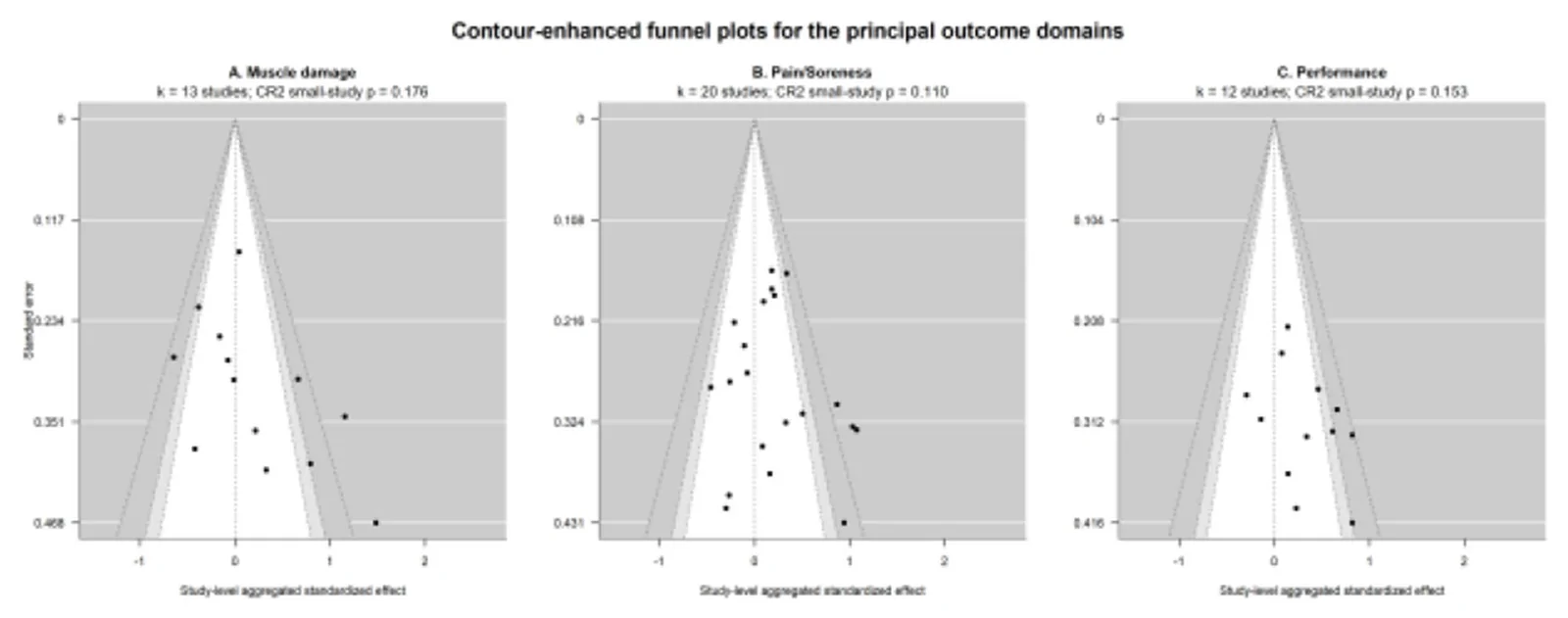
Contour-enhanced funnel plots for the principal outcome domains: **(A)** Muscle damage; **(B)** Pain/Soreness; and **(C)** Performance.

The GRADE certainty of evidence assessments were conducted based on the primary effect estimates, outcome-specific RoB 2 judgments, domain-specific heterogeneity and prediction intervals, optimal information size (OIS), and domain-specific small-study effect diagnostics for each primary outcome domain. The predefined OIS for all outcome domains was 785 participants, and the actual number of included participants fell below this threshold in all domains. The Muscle damage domain included 13 studies with 320 participants, and the certainty of evidence was rated as Low, downgraded by one level each for inconsistency and imprecision. The Pain/Soreness domain included 20 studies with 459 participants, and the certainty of evidence was rated as Very low, downgraded by one level each for risk of bias, inconsistency, and imprecision. The Perceptual domain was contributed by only 1 study with 33 participants, and the certainty of evidence was rated as Very low, downgraded by one level for risk of bias and by two levels for very serious imprecision; due to the single-study nature, between-study heterogeneity and formal publication bias tests could not be reliably assessed. The Performance domain included 12 studies with 249 participants, and the certainty of evidence was rated as Low, downgraded by one level each for risk of bias and imprecision. Across the Muscle damage, Pain/Soreness, and Performance domains where domain-specific small-study effect tests could be performed, no clear evidence of small-study effects was found; the corresponding domain-specific contour-enhanced funnel plots are displayed in Figure 4. Complete GRADE domain-specific judgment rationales and the Summary of Findings are presented in Table 4, while complete publication bias diagnostics and sensitivity analysis results are provided in Supplementary File S4.

**Table 4:** Summary of findings and certainty of evidence.

Outcome domain	Studies	Effect sizes	Participants	Effect estimate (95% CI)	Risk of bias	Inconsistency	Indirectness	Imprecision	Publication bias	Certainty
Muscle damage	13	65	320	0.097 (−0.249, 0.442)	Not serious	Serious	Not serious	Serious	Not detected	Low
Pain/Soreness	20	83	459	0.252 (0.049, 0.454)	Serious	Serious	Not serious	Serious	Not detected	Very low
Perceptual	1	6	33	1.171^†^	Serious	Not estimable/no downgrade	Not serious	Very serious	Not formally testable/no downgrade	Very low
Performance	12	41	249	0.238 (−0.017, 0.493)	Serious	Not serious	Not serious	Serious	Not detected	Low

Treatment-effect estimates were taken directly from the principal outcome-domain analyses based on the locked primary dataset of unadjusted post-exercise endpoint values. All included evidence originated from randomized controlled trials and therefore started at High certainty. Certainty was downgraded according to the five GRADE domains: risk of bias, inconsistency, indirectness, imprecision, and publication bias. “Serious” indicates a one-level downgrade and “Very serious” indicates a two-level downgrade. The prespecified optimal information size (OIS) was 785 participants per outcome domain, based on a target standardized mean difference of 0.20, a two-sided α of 0.05, and 80% power; none of the outcome domains met the OIS. Positive effect estimates favor polyphenol supplementation.^†^The Perceptual estimate was contributed by one independent study and is descriptive only; CR2 inferential statistics, heterogeneity, and prediction intervals were not estimable, and publication bias could not be formally assessed.

## 4 Discussion

### 4.1 Summary of main findings

After standardizing the primary effect size estimands and explicitly accounting for correlated effect sizes within the same study, the interpretation of this study should focus on outcome domain analyses rather than overall composite effects across different outcomes. The existing evidence presents a relatively clear separation between “subjective symptoms and objective recovery”: polyphenol supplementation may provide a small alleviation of post-exercise pain/soreness, but current evidence is insufficient to support its ability to consistently improve biochemical markers of muscle damage or muscle function and athletic performance. Because the Perceptual domain was supported by only a single independent study, it cannot be inferred that this category of subjective perception outcomes possesses a larger or more definitive intervention effect. Consequently, a more appropriate interpretation is that polyphenol supplementation may improve certain subjective recovery experiences post-exercise, but this symptom-level improvement should not be directly equated with reduced muscle damage or accelerated functional recovery. The overall limited certainty of evidence across outcome domains also calls for caution regarding the magnitude of effects and their generalizability.

Analyses of recovery time and supplementation strategies further constrain more specific practical inferences. Current data failed to identify statistically detectable effect differences among predefined recovery time windows; however, this neither proves that the intervention effects remain temporally consistent nor rules out the presence of peak windows that went unrecognized due to limited study numbers and statistical power. Similarly, although point estimates varied across supplementation strategies, overall comparisons did not provide sufficient evidence to demonstrate that any single strategy—whether pre-loading, acute, or peri-exercise supplementation—was superior to the others. Therefore, optimal supplementation timing or recovery windows cannot be definitively determined at this stage.

Although the overall composite effect derived across biochemical, subjective, and functional outcomes can be used to describe the general direction, because it pools outcomes with distinct clinical meanings, this result should continue to be regarded as secondary/exploratory evidence rather than replacing outcome domain-specific primary conclusions. Sensitivity analyses generally showed that the direction of effect estimates remained relatively stable, though statistical significance exhibited some dependence on certain analytical specifications. Consequently, these results further restrict strong interpretations of the cross-outcome composite effect, without altering the principle that outcome domain-specific analyses should serve as the primary basis for clinical inferences.

### 4.2 Interpretation of outcome-specific effects

The most important outcome-level insight of this study is not that polyphenols exerted a uniform effect on “overall recovery,” but rather that the physiological and clinical constructs reflected by different recovery indicators are not equivalent. Pain/Soreness reflects the subjective experience of post-exercise pain and discomfort, whereas biochemical markers of muscle damage and Performance reflect circulation-related damage indicators and functional recovery, respectively (1). Existing evidence leans toward supporting the notion that polyphenols may alleviate post-exercise pain/soreness experiences, but it cannot be inferred that they simultaneously mitigate muscle tissue damage or accelerate functional recovery. Therefore, a conceptual distinction must be maintained between subjective symptom improvements and objective recovery indicators; changes in one outcome type should not be extrapolated to imply improvement across the entire recovery process.

The potential benefits for Pain/Soreness possess a degree of biological plausibility. Post-exercise inflammatory responses, redox imbalance, and nociception-related processes may contribute to the development of post-exercise pain and soreness, whereas plant-derived polyphenols may influence this process by modulating inflammatory signaling and cellular redox homeostasis (1, 4). However, this meta-analysis did not directly test these mechanisms; thus, this interpretation should be viewed as a plausible biological explanation for the observed results rather than confirmed mechanisms. More importantly, pain/soreness is a subjectively reported outcome, and its measurement may also be influenced by scale characteristics, exercise models, measurement contexts, and individual perceptual differences. Combined with the low certainty of evidence in this domain, the most appropriate current conclusion remains that polyphenols may provide limited symptom relief rather than definitively promoting overall muscle recovery.

The uncertainty within the Muscle damage domain further illustrates that circulating biochemical markers cannot be simply regarded as direct surrogate indicators of muscle structural repair. Circulating indices such as CK, LDH, and myoglobin are associated with post-exercise changes in muscle cell membrane permeability and intracellular protein leakage, but their fluctuations are also influenced by multiple factors including exercise type and intensity, training and individual characteristics, sampling timing, and pre-analytical factors (1, 57). Consequently, even if a nutritional intervention can modulate post-exercise inflammatory or oxidative environments, it does not necessarily produce consistent changes in circulating markers; conversely, the lack of stable improvements in biochemical markers alone cannot prove the absence of any biological effects. Based on current evidence, a safer interpretation is that there is insufficient definitive evidence indicating that polyphenols consistently improve biochemical markers of muscle damage following EIMD, and it should not be further inferred whether they have or have not promoted muscle structural repair.

The Performance domain similarly requires differentiation from symptomatic recovery. Functional outcomes such as strength, jumping, and power occupy a more downstream position in the recovery process, and their recovery is influenced not only by local muscle status but also by multiple links including force transmission, excitation-contraction coupling, neuromuscular function, and overall fatigue states (1, 2). Therefore, pain reduction does not necessarily translate into concurrent improvements in strength or athletic performance. Furthermore, the current discrepancy between subjective symptoms and functional outcomes cannot be interpreted as a temporal sequence of “perceptual recovery preceding functional recovery,” because this study did not establish longitudinal causal relationships among these outcomes, nor is there evidence demonstrating that the two categories of outcomes possess distinct recovery time trajectories. Similarly, the Perceptual domain was contributed by only a single independent study, and its descriptive estimate cannot be used to prove that this domain exhibits a larger effect than other outcomes, nor can it serve as a basis for comparing superiority among outcome domains.

Overall, the differences among outcome domains strongly support viewing EIMD recovery as a multidimensional process rather than a unified construct that can be adequately represented by a single indicator or a cross-domain overall effect. For the present study, the signal of highest clinical interpretive value is concentrated in Pain/Soreness, though its certainty of evidence remains limited; regarding biochemical markers of muscle damage and athletic performance, current evidence is still insufficient to support stable benefits. Such domain-specific interpretations also illustrate that the overall composite effect formed across biochemical, subjective, and functional indices can only serve to provide supplementary overall directional information, rather than replacing the evidence of the primary outcome domains themselves.

### 4.3 Heterogeneity and temporal uncertainty

Different outcome domains exhibited varying patterns of heterogeneity, which further illustrates that EIMD recovery is not a homogeneous process that can be fully summarized by a single overall effect. Effect variations were relatively more pronounced in Muscle damage and Pain/Soreness, whereas heterogeneity was lower in Performance; however, lower heterogeneity in itself does not imply more definitive evidence. Primary studies varied in terms of exercise injury models, participant training status, polyphenol sources and formulations, specific measurement indicators, and follow-up timing, all of which may have contributed to the observed variance (4, 8); however, because this study did not conduct exhaustive and independent moderator tests for all potential sources, heterogeneity cannot be attributed to any single specific factor. Based on this evidence structure, heterogeneity should be interpreted within each outcome domain rather than substituting domain-specific judgments with an overall composite model spanning biochemical, subjective, and functional outcomes.

The temporal dimension should likewise be understood through the lens of “uncertainty” rather than a “stable trajectory.” The failure to detect statistically distinguishable effect differences among predefined recovery time windows can only indicate that existing data are insufficient to differentiate intervention effects across distinct temporal stages; it does not prove that polyphenol actions remain consistent throughout the recovery period, nor does it imply the absence of peak effects. In particular, discrete temporal groupings compress variations within the same time window, and the uneven distribution of study counts contributed by different time points may both reduce the capacity to discern true temporal differences. Therefore, favorable estimates appearing in a single time window or certain morphological patterns emerging from exploratory temporal models should not override the predefined overall time moderator tests.

Consequently, current evidence neither determines an optimal recovery time window nor rules out the possibility that true effects vary across recovery stages. A more appropriate interpretation is that the temporal resolution and statistical information volume of existing studies are still inadequate to reliably characterize the recovery time trajectory of polyphenol interventions; any statements regarding “sustained efficacy,” “temporal consistency,” or “the absence of distinct peaks” exceed the scope of what the existing evidence can support.

### 4.4 Supplementation strategy and practical interpretation

Existing evidence is insufficient to support a clear supplementation timing for polyphenols that is superior to other regimens. Although effect estimates varied numerically across supplementation strategies, overall comparisons between strategies failed to establish a statistically superior relationship, and the number of studies and intervention protocols underlying each strategy were uneven. Therefore, the larger point estimate for pre-loading should not be interpreted as possessing a definitive biological or clinical advantage, nor should the uncertainty surrounding acute or peri-exercise results be equated with ineffectiveness of these strategies. Based on current evidence, the optimal initiation time, supplementation duration, or optimal timing cannot be determined, and it is even less appropriate to formulate clinical recommendations prioritizing a specific supplementation strategy based on this. The final overall strategy test similarly failed to support clear differences among the different strategies.

From the perspective of sports medicine and clinical practice, polyphenol supplementation is more appropriately viewed as an adjunctive nutritional intervention that may improve the post-exercise pain/soreness experience, rather than a therapeutic modality promoting the entire EIMD recovery process (58, 59). The alleviation of pain/soreness itself may hold practical value, particularly when symptoms affect comfort or subjective recovery experience; however, existing evidence has not demonstrated that this improvement consistently translates into faster recovery of muscle function, enhanced athletic performance, or reduced biochemical markers of muscle damage. Therefore, subjective symptom improvement cannot serve as surrogate evidence for the restoration of training capacity, complete functional recovery, or accelerated tissue repair. Given that the certainty of evidence for pain/soreness remains very low, caution should also be maintained regarding the magnitude of its actual clinical benefits.

This boundary is particularly important in a medical context. This review investigates EIMD occurring in healthy adults following exercise, rather than acute muscle tears, tendon injuries, or other clinical musculoskeletal disorders. Therefore, the current results cannot be extrapolated to suggest that polyphenols can treat sports injuries, shorten clinical rehabilitation times, or support early return to play. Similarly, this study has not established optimal dosages, formulations, or supplementation durations that can be applied at a prescription level. For healthy athletic populations choosing to use polyphenols, their rational positioning should be as an optional auxiliary measure within comprehensive recovery management, rather than a replacement for load management, adequate nutrition, sleep, and other established recovery measures (60, 61).

Consequently, current evidence supports a relatively limited yet clinically clearer positioning: polyphenols may help some healthy individuals reduce post-exercise pain and soreness, but there is still insufficient evidence to prove that they universally accelerate objective recovery, nor is there evidence determining the optimal supplementation timing. Clinical and practical decisions should revolve around this uncertainty rather than forming specific supplementation recommendations based on numerical advantages in a single subgroup.

### 4.5 Strengths, limitations, and research priorities

The primary strength of this study lies in maintaining consistency among the research question, effect size definition, and statistical inference to the greatest extent possible. The primary analysis was restricted to unadjusted post-exercise endpoint values, avoiding the mixed interpretation of endpoint values, absolute change values, and percentage change values as the same estimand. Concurrently, a three-level model was employed to retain multiple correlated outcomes and time points within the same study, and effect size dependencies were handled through a structured variance-covariance matrix and study-level CR2 inference. For crossover trials and multi-arm trials with shared controls, effect sizes and covariance treatments matching their respective designs were adopted. Furthermore, risk of bias and GRADE certainty were evaluated at the outcome-specific level, and the sensitivity of primary inferences to analytical specifications was tested through sensitivity analyses across different study designs, risks of bias, data extraction methods, correlation parameters, and covariance structures. These measures enhanced the internal consistency and reproducibility of the complex repeated-measures evidence synthesis. In addition, the final analyses also implemented supplementary analyses—such as estimand separation, deterministic single-effect selection, influence diagnostics, alternative temporal classifications, and shared-control structure validation—to further evaluate the sensitivity of primary inferences to various analytical settings.

These methodological improvements cannot eliminate the inherent limitations of the original evidence itself. First, the certainty of evidence for each primary outcome domain was low or very low, and the available information volume was insufficient, leaving noticeable uncertainty regarding effect magnitudes and generalizability; the perceptual domain, in particular, was contributed by only a single independent study, precluding the evaluation of between-study consistency. Second, the included trials exhibited substantial clinical and methodological variations in polyphenol sources, formulations, and supplementation protocols, EIMD induction methods, training backgrounds, outcome measurements, and follow-up arrangements. These differences broadened the research contexts covered by the existing evidence while simultaneously limiting the ability to draw precise conclusions regarding specific compounds, doses, or populations. The small number of independent studies underlying certain domains and supplementation strategies also reduced the capacity of moderator analyses, publication bias diagnostics, and subgroup comparisons to discern true differences.

Third, certain methodological parameters still relied on plausible assumptions. Original studies typically failed to report the correlations between treatment-condition observations in crossover designs, and the covariance structures of multiple correlated effect sizes within studies could not be directly obtained from all original reports. Although this study conducted systematic sensitivity analyses using various correlation parameters and structured covariance settings, this could only test the sensitivity of conclusions to a plausible range of assumptions rather than recovering the true correlation structures unmeasured or unreported by the original studies. In addition, some numerical values were derived via graphical digital extraction, and statistical inferences exhibited some variation under alternative analytical settings. Therefore, the understanding of evidence robustness should emphasize effect directions and overall evidence patterns rather than relying solely on statistical significance within a single model.

Future research should prioritize addressing the aforementioned evidence gaps rather than continuing to add highly heterogeneous, small-sample trials. First, there is a need for adequately powered, pre-registered randomized controlled trials utilizing consistent core outcome sets, while systematically reporting pain/soreness, muscle damage indicators, and functional performance to avoid representing overall recovery with a single metric. Second, denser and more standardized post-exercise follow-ups should be adopted to support continuous temporal or predefined time interaction analyses, thereby truly testing whether clinically meaningful recovery windows exist. Third, regarding supplementation strategies and formulation selection, direct head-to-head comparisons of dosage, duration, and supplementation timing are urgently needed under identical population, exercise model, and polyphenol formulation conditions, rather than relying on indirect subgroup comparisons across different studies. Finally, future crossover trials should comprehensively report treatment-condition correlations, period effects, and potential carryover effects, and multi-arm trials should provide sufficient arm-specific information to reduce the reliance of subsequent evidence synthesis on covariance assumptions.

## 5 Conclusion

This systematic review and three-level meta-analysis indicate that following exercise-induced muscle damage (EIMD) in healthy adults, plant-derived polyphenol supplementation may exert a small favorable effect on post-exercise pain and soreness, but currently there is insufficient evidence to demonstrate that it can consistently improve biochemical markers of muscle damage or accelerate the recovery of muscle function and athletic performance. Perceptual outcomes were supported by evidence from only a single study, from which definitive conclusions cannot be drawn. Existing research is also insufficient to determine optimal recovery time windows or to prove that any single supplementation strategy is superior to others. Given that the certainty of evidence for all primary outcome domains is low or very low, polyphenols are more appropriately regarded as an adjunctive nutritional measure that may alleviate post-exercise subjective discomfort, rather than an intervention capable of universally promoting overall recovery following EIMD. Future randomized controlled trials with adequate sample sizes, pre-registration, standardized outcomes, and denser follow-ups are needed to clarify their true clinical effects and appropriate supplementation regimens.

## Data Availability

The original contributions presented in the study are included in the article/supplementary material, further inquiries can be directed to the corresponding author.
